# Gene set analysis for time-to-event outcome: comparison of a new approach based on the generalized Berk–Jones statistic with existing methods in presence of intra gene-set correlation

**DOI:** 10.1093/bib/bbag262

**Published:** 2026-05-31

**Authors:** Thomas Ferté, Laura Villain, Rodolphe Thiébaut, Boris P Hejblum

**Affiliations:** SISTM team, Inserm Bordeaux Population Health Research Center, UMR 1219, Univ. Bordeaux, 146 rue Léo Saignat, F-33000 Bordeaux, Gironde, France; SISTM team, Centre INRIA de l'Université de Bordeaux, 200 Av. de la Vieille Tour, F-33400 Talence, Gironde, France; VRI, Vaccine Research Institute, Hôpital Henri Mondor, 8 Rue du Général Sarrail, 94000 Créteil, F-94000 Créteil, Val-de-Marne, France; Pôle de Santé Publique, CHU de Bordeaux, Pl. Amélie Raba Léon, F-33000 Bordeaux, France; SISTM team, Inserm Bordeaux Population Health Research Center, UMR 1219, Univ. Bordeaux, 146 rue Léo Saignat, F-33000 Bordeaux, Gironde, France; SISTM team, Centre INRIA de l'Université de Bordeaux, 200 Av. de la Vieille Tour, F-33400 Talence, Gironde, France; VRI, Vaccine Research Institute, Hôpital Henri Mondor, 8 Rue du Général Sarrail, 94000 Créteil, F-94000 Créteil, Val-de-Marne, France; ESQlabs GmbH, Am Sportplatz 7, D-26683 Saterland, Niedersachsen, Germany; SISTM team, Inserm Bordeaux Population Health Research Center, UMR 1219, Univ. Bordeaux, 146 rue Léo Saignat, F-33000 Bordeaux, Gironde, France; SISTM team, Centre INRIA de l'Université de Bordeaux, 200 Av. de la Vieille Tour, F-33400 Talence, Gironde, France; VRI, Vaccine Research Institute, Hôpital Henri Mondor, 8 Rue du Général Sarrail, 94000 Créteil, F-94000 Créteil, Val-de-Marne, France; Pôle de Santé Publique, CHU de Bordeaux, Pl. Amélie Raba Léon, F-33000 Bordeaux, France; SISTM team, Inserm Bordeaux Population Health Research Center, UMR 1219, Univ. Bordeaux, 146 rue Léo Saignat, F-33000 Bordeaux, Gironde, France; SISTM team, Centre INRIA de l'Université de Bordeaux, 200 Av. de la Vieille Tour, F-33400 Talence, Gironde, France; VRI, Vaccine Research Institute, Hôpital Henri Mondor, 8 Rue du Général Sarrail, 94000 Créteil, F-94000 Créteil, Val-de-Marne, France

**Keywords:** gene set analysis, survival analysis, Generalized Berk–Jones statistics, Cox proportional hazards model, gliomas, breast cancer

## Abstract

Gene set analysis evaluates the collective impact of groups of genes on an outcome of interest, such as disease occurrence. By incorporating biological knowledge through predefined gene sets, this approach enhances the interpretability of results and improves statistical power compared with gene-wise analyses. In the context of time-to-event data, existing methods are limited and fail to account for potentially strong correlations within gene sets. Given the strong performance of the Generalized Berk-Jones (GBJ) statistic, which effectively incorporates correlation within the test statistic, we adapted this method to the time-to-event framework using a Cox model. We then compared its performance with established methods, including the Cauchy, Harmonic Mean, Wald test, global test, and global boost test. We further benchmarked these methods in two different real-world datasets: gliomas and breast cancer. Our proposed method, sGBJ, shows an overcontrol of Type I error, leading to reduced statistical power compared with other methods in numerical studies particularly when the number of genes is greater than or equal to the number of observations. The Wald test and global boost test generally exhibited the highest power, except in very high-correlation settings for the global boost test, while the Wald test could not adjust for confounders in current implementations.

## Introduction

The analysis of the whole transcriptome due to RNA-seq [1] is usually based on differential expression methods. Statistical methods such as edgeR [2], DESeq2 [3], limma-voom [4], or dearseq [5] allow comparing gene expression between groups of patients or within patients over time. However, gene-wise analysis exhibits several limitations: (i) the high-dimensionality of gene expression data (often tens of thousands of genes are measured per sample) might lead to either no gene being detected differentially expressed after multiple testing correction, or on the contrary to a very large list of genes that is difficult to interpret biologically; (ii) many genes interact within biological pathways, but with potentially only small individual changes affecting a few genes, genes within a pathway can fail to be detected as differentially expressed. To address these issues, Gene Set Analysis leverages predefined sets of genes—available in databases such as MSigDB [6], KEGG [7], or Gene Ontology [8] for instance—and identifies groups of genes differentially expressed rather than single genes. The reduction of the number of statistical tests and the strength of a coordinated signal within a gene set both increase statistical power, and avoid missing important biological links with the outcome of interest. Numerous methods have been proposed to analyze gene set expression in various context [9], such as Gene Set Enrichment Analysis (GSEA) [10], GSA [11], Time-course Gene Set Analysis (TcGSA) [12], Generalized Higher Criticism (GHC) [13], dearseq [14], or the Generalized Berk–Jones (GBJ) statistic [15].

In the time-to-event context, some methods are able to tackle the high-dimensionality of gene expression data, such as survival random forest [16] or penalized Cox regression [17], but only a handful can perform gene set analysis. Based on Kernel Machine, Cai *et al*. [18] proposed a score test for survival gene set analysis, which Neykov *et al*. [19] later adapted to take into account competing risk. Besides, three additional methods are building on the Cox model to perform survival gene set analysis, using different test statistics and relying on permutations to compute the associated $P$-values: (i) the global test [20], (ii) the Wald test [21], and (iii) the global boost test [22]. In their review, Lee *et al*. [23] found all three tests outperformed GSEA in terms of statistical power, even though the global test relies on the strong hypothesis that all regression coefficients are sampled from one unique probability distribution, and none of the three compared tests takes into account the correlation structures between the genes in the pathway.

Gaynor *et al*. [15] compared the GBJ statistic with GSA, GSEA, and GHC to identify gene sets differentially expressed between two tumor grade, and they concluded that their proposed GBJ method was more consistent. The GBJ statistic has the advantage of not requiring any distributional assumption on the count data or the regression coefficients, while also taking into account the correlation structure between the genes within the same set. We propose to adapt this GBJ statistic in the time-to-event context using a statistic derived from the Cox model. We denote our method by sGBJ for “survival Generalized Berk–Jones.”

In this paper, we first describe our new method sGBJ in section Method before benchmarking existing methods together with sGBJ in a simulation study inspired by both Lee *et al*. [23] and real-world data. We also compare the results of the different methods in two real-world data analyses, with brain cancer data and breast cancer data, respectively, in section Real-world medical data applications, and finally discuss our results and their limits in section Discussion. The sGBJ method is available as an R package from CRAN at https://CRAN.R-project.org/package=sGBJ.

## Method

### The Generalized Berk–Jones set-based testing method

The GBJ statistic can be used in a set-based testing procedure to determine the association between gene expression in a gene set and a given clinical outcome (e.g. tumor grade). Introduced by Sun *et al*. [24], it was used in the context of Genome-Wide Association Studies (GWAS) [25] and its consistency was evaluated for identifying pathways whose expression is associated with either low or high grade of breast cancer [15]. Below is a four-step description of this GBJ testing procedure:

1. Model and null hypothesis specification for a given gene set of $d$ genes: Sun *et al*. [24] study the association between the outcome of interest of patient $i$, denoted, $Y_{i}$ and the expression of $d$ genes, denoted $\mathbf{G}_{i}$ with a generalized linear model $g(\mathbf{\mu _{i}})=\mathbf{X}_{i}^{\mathbf{\alpha }} + \mathbf{G}_{i}^{\mathbf{\beta }}$, where $\mathbf{\mu _{i}}$ denotes the conditional mean of $Y_{i}$ and $\mathbf{X}_{i}$ denotes the other covariates.
2. For each gene $j$ of a given gene set with $j = \{1,..., d\}$, a score value $Z_{j}$ is computed. It must verify $\mathbf{Z} \sim \mathcal{N}(\mathbf{0},\mathbf{\Sigma })$ under the null hypothesis.
3. The GBJ statistic is computed for the whole gene set based on the $Z_{j}$ values of each gene, using the threshold function $S$ computing the number of genes in the gene set that have an absolute value of their score $Z_{j}$ higher than a limit $t$ and thus defined as 
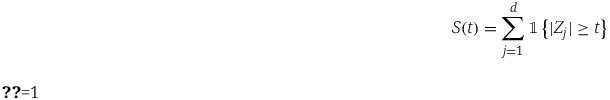
.
4. Once the GBJ statistic is computed, its associated $P$-values are determined with a root-finding algorithm that identifies the boundaries points $b_{j}$, i.e. the limit value of the $j$th greatest absolute value of $\mathbf{Z}$.

### Extension to time-to-event data

The GBJ method as defined by Sun *et al*. [24] can be applied to any generalized linear model, but not for time-to-event data analysis. From the third step onward, the procedure relies on a score statistic calculated for each gene within the gene set, with a multivariate normal distribution assumption on this score centered around zero under the null hypothesis (i.e. no association between gene expression and the dependent time-to-event variable $\boldsymbol{Y}$). We propose a survival GBJ method, named sGBJ, that uses a score suited to time-to-event in order to compute a GBJ statistic in this context.

#### Model

To deal with a time-to-event outcome, we rely on a Cox proportional hazards regression model:


(1) \begin{eqnarray*}& \lambda(t,\mathbf{G}_{i})=\lambda_{0}(t)\mathrm{exp}(\mathbf{G}_{i}^{\boldsymbol{\beta}}),\end{eqnarray*}

with $\mathbf{G}_{i}$ being the vector of gene expressions of patient $i$. The null hypothesis is that there is no association between patient survival and the expression of genes within the set: $H_{0}: \boldsymbol{\beta} = \mathbf{0}_{d\times 1}$.

#### Z score

For each gene $j$ of the gene set, we compute the value $Z_{j}$ as the square root of the Wald statistic [26]:


(2) \begin{eqnarray*}& Z_{j}=\sqrt{W_{j}}=\frac{\widehat{\beta_{j}}-\beta_{0}}{se(\widehat{\beta_{j}})}= \frac{\widehat{\beta_{j}}}{se(\widehat{\beta_{j}})}.\end{eqnarray*}

This yields a vector $\mathbf{Z}$ that follows $\mathcal{N}(\mathbf{0},\mathbf{\Sigma })$ under the null hypothesis $H_{0}$. $\mathbf{\Sigma }$ is estimated through permutations of the original survival observations: for each permutation $p \in \{1,\dots , P\}$, the $Z^{*(p)}_{j}$ value for each gene $j$ gives us a $\mathbf{Z}^{*(p)}$ vector of length $d$, allowing us to estimate $\Sigma$ with the empirical covariance across permutations $\widehat{\mathbf{\Sigma }}$.

#### GBJ statistic

The GBJ statistic is computed according to Sun *et al*. [24]:


(3) \begin{eqnarray*}& \mathcal{G}_{d} = \max_{\{1 \geq j \geq \frac{d}{2}\}} \mathrm{log} \left[ \frac{P_\mu}{P_{0}} \right]{\mathbb{1}}_{\left\{2 \phi(|Z|_{(d-j+1)}) < \frac{j}{d} \right\}},\end{eqnarray*}

where


(4) \begin{eqnarray*} P_\mu &= \mathrm{Pr}\left\{S(|Z|_{(d-j+1)})=j|E(\mathbf{Z})=\widehat{\mu}_{jd}\cdot{\mathbf{J}}_{d},cov(\mathbf{Z})=\mathbf{\widehat{\Sigma}} \right\} \end{eqnarray*}

(5) \begin{eqnarray*} P_{0} &= \mathrm{Pr}\left\{S(|Z|_{(d-j+1)})=j|E(\mathbf{Z})=0\cdot{\mathbf{J}}_{d},cov(\mathbf{Z})=\mathbf{\widehat{\Sigma}} \right\} \end{eqnarray*}

with ${\mathbf{J}}_{d}^{T}=(1,1,...,1)_{d\times 1}$, $\phi$ the survival function of a standard normal random variable, and $\widehat{\mu }_{jd}>0$ solving the following equation:


(6) \begin{eqnarray*} & \frac{j}{d} = 1 - \left\{ \phi(|Z|_{(d-j+1)}-\widehat{\mu}_{jd}) - \phi(- |Z|_{(d-j+1)}-\widehat{\mu}_{jd}) \right\}.\end{eqnarray*}

Of note, the GBJ statistic $\mathcal{G}_{d}$ is calculated only on the upper half of the ordered values of $|\mathbf{Z}|$, as thresholds in the central part of the distribution are uninformative for distinguishing the null from the alternative, and can be represented as the maximum of a set of likelihood ratios on $S(t)$ [25].

#### $P$ -value computation

The $P$-value can be computed as


(7) \begin{eqnarray*}& \mathrm{Pr}\left(\mathcal{G}_{d} \geq \widehat{\mathcal{G}}_{d}\right)=1- \mathrm{Pr} \left\{ \forall j=1,..,d: |Z_{j}| \leq b_{j} | \mathbf{Z} \sim \mathcal{N}(\mathbf{0},\mathbf{\Sigma}) \right\},\end{eqnarray*}

with $\widehat{\mathcal{G}}_{d}$ the observed value of the GBJ statistic. The associated rejection region is then


(8) \begin{eqnarray*} \mathrm{Pr} &\left\{ \forall j: |Z_{j}| \leq b_{j} | \mathbf{Z} \sim \mathcal{N}(\mathbf{0},\mathbf{\Sigma}) \right\} \nonumber\\ &= \mathrm{Pr} \left\{ \forall j: S(b_{j}) \leq (d-j) | \mathbf{Z} \sim \mathcal{N}(\mathbf{0},\mathbf{\Sigma}) \right\}.\end{eqnarray*}

## Numerical studies and comparisons

### Numerical simulation scenarios

sGBJ does not require any distributional assumption on the gene expression matrix, thus we simulated this matrix for $N \in \{50, 100\}$ observations following a multivariate normal distribution $\mathcal{N}(\mathbf{0},\mathbf{C})$, with $\mathbf{C}$ being the gene covariance matrix. Similarly to Lee *et al*. [23], we simulated a gene set of $NG = \{10, 50, 100\}$ genes, among which $20\%$ were significantly associated with survival. We set the variance $C_{jj}=0.2$ for each gene $j$. We generated three different correlations scenarios designed to mimic observed features of real-world data inspired by both Rembrandt [27] and a breast cancer study [28] datasets as depicted in the supplementary materials section 1:

- **Case (I)**: overall correlation follows a nonstandard beta $corr(G_{j}, G_{j^{\prime}}) \sim NSBeta(20, 20, min=-1, max =1)\,\forall j\neq j^{\prime}$
- **Case (II)**: this scenario mimics both Rembrandt and Breats Cancer data (see section Survival analysis in glioma subtypes and Progression-free survival in breast cancer).
  (i) correlation between significant genes follows a $NSBeta(10, 10, min=-1, max =1)$,
  (ii) correlation with non-significant genes follows a $NSBeta(25, 25, min=-1, max =1)$
- **Case (III)**:
  (i) correlation between significant genes is $0.2$,
  (ii) correlation with non significant genes is $0$.

Note that Cases (I) and (II) are not guaranteed to generate positive definite matrix. So once the correlation values are sampled, if the matrix is not positive definite, the nearest positive definite matrix is computed using the algorithm defined in Higham [29].

Survival times were then generated using a Cox model featuring an effect $\beta$ for the significant genes. We investigated three different effect types:

- **Type (A)**: $\beta _{j} \sim \mathcal{N}(0, 0.4^{2})$.
- **Type (B)**: half of the gene effects follow $\beta _{j} \sim \mathcal{N}(-0.4, 0.2^{2})$, while the other half follow $\beta _{j} \sim \mathcal{N}(0.4, 0.2^{2})$. This setting mimics the Rembrandt dataset (see section Survival analysis in glioma subtypes).
- **Type (C)**: half of the gene effects follow $\beta _{j} \sim \mathcal{N}(-0.8, 0.4^{2})$, while the other half follow $\beta _{j} \sim \mathcal{N}(0.8, 0.4^{2})$. This setting mimics breast cancer data (see section Progression-free survival in breast cancer).
- **Type (Z)**: $\boldsymbol{\beta} = \mathbf{0}$, evaluates the type-I error

In all scenarios, the correlation matrix of $\boldsymbol{\beta}$ is the same as the correlation matrix of $\mathbf{G}$. We also considered potential censoring: censoring times were generated following an exponential distribution for $30\%$ of the observations.

For Types A, B, and C, $500$ independent Monte-Carlo repetitions were generated. For Type Z, $2000$ independent Monte-Carlo repetitions were generated.

### Compared methods

sGBJ was compared with five state-of-the-art methods: (i) the global test [20], (ii) the Wald test [21], (iii) the global boost test [22], (iv) the Cauchy association test, and the (v) Harmonic mean $P$-value, which are also based on statistics derived from the Cox model. The global test relies on the hypothesis that all $\beta _{j}$ are sampled from the same normal distribution centered in $0$ with a common variance $\sigma ^{2}$, so the null hypothesis can be reduced to testing $\sigma ^{2}=0$. The Wald test uses the sum of squares of the Wald statistics from a Cox model applied individually to each gene within a gene set, possibly adjusted on other covariates. The global boost test combines the Cox model with a boosting algorithm to assess the additional predictive value of gene expression within a gene set. The Cauchy and Harmonic Mean tests rely on $P$-value combination approaches [30–32]. $P$-values for the global test, Wald test, and global boost test are computed using permutation procedures, according to currently available implementations.

### Results

**Figure 1:**
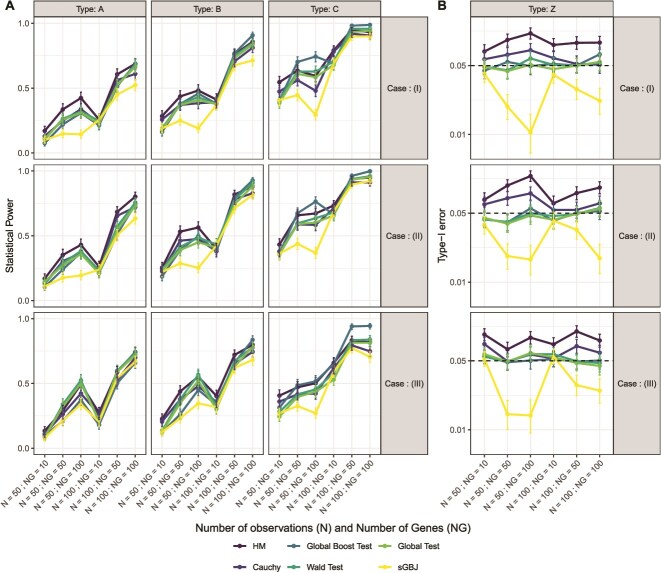
Panel **A** shows the statistical power of the six methods (sGBJ, Global Boost Test, Wald Test, Global Test, Cauchy, and Harmonic Mean) across nine different scenarios, which combine three correlation cases (I, II, and III) with three types of effects (A, B, and C — more details on these scenarios are provided in Section Numerical simulation scenarios); panel **B** presents the type I error rates of the methods, stratified by the different correlation cases.

Figure 1 highlights the performance of the six methods across the different simulation settings. Panel B shows that the Cauchy and Harmonic Mean methods failed to control Type-I error, whereas sGBJ tended to overcontrol it. This phenomenon was more pronounced as the number of genes increased, particularly when the number of observations was small, and it diminished when the number of observations was large (see Supplementary Section 3.3, with 1000 observations). Off note, QQ-plot of the $P$-value distribution available in supplementary section 2 showed that sGBJ had a high number of $P$-values close to 1, which might partly explain the overconservative Type-I error control. Wald test, global test, and global boost test controlled correctly for Type-I error.

Panel A reveals that statistical power varied significantly depending on the scenario, with lower power observed as the number of individuals or genes decreased. Overall, the Wald test, global test, and global boost test exhibit comparable power. In the Type C scenario, the global boost test showed a slight advantage, while the global test performed slightly worse, likely because its assumption of normally distributed coefficients was violated. The sGBJ method achieves power comparable with the other methods when the number of genes is smaller than the number of observations, but shows reduced power when the number of genes equals or exceeds the number of observations, reflecting challenges in high-dimensional settings. This loss of power is largely attributable to its overconservative control of the Type I error.

### Sensitivity analysis

We investigated the effect of the number of permutations used by sGBJ on the estimation of the gene-effect covariance matrix (see Section 4 of the Supplementary Materials). The results show a sharp improvement in estimation accuracy between 8 and 200 permutations, followed by convergence beyond 200 permutations, with minimal gains up to 5000. Based on this empirical stability, we recommend using 1000 permutations as a practical compromise between estimation accuracy and computational cost.

We performed a sensitivity analysis exploring the effect of sparser gene effects, where only $5\%$ or $10\%$ of genes were significant. This analysis is available in Supplementary Section 3.2. Overall, the results are similar to the main analysis, with sGBJ showing conservative Type I-error control. As expected, this results in a more pronounced loss of power when the proportion of significant genes is small (e.g. $5\%$).

We also investigated additional scenario in which the correlation between significant genes was 0.9 to assess the behavior of the methods under an extreme condition. Detailed results are provided in Section 3.1 of the Supplementary Materials. In this setting, the power of the different methods remained stable across varying numbers of genes and sample sizes, and sGBJ exhibited robust performance with conservative Type I error. In contrast, the Global Boost Test showed a decrease in power under strong correlation, while the Harmonic Mean and Cauchy combination methods continued to fail to control Type I error.

## Real-world medical data applications

### Survival analysis in glioma subtypes

Glioma represent over 80% of malignant brain tumor, and are associated with poor survival with <5% of survival at 5 years for the glioblastoma (the most common type of glioma) [33]. Glioma can be classified to different types according to the World Health Organization (WHO), with mostly histological and molecular alterations criteria (a first classification from 2007 was revised in 2016). The severity can increase from grade I to grade IV, grade IV being mostly the glioblastoma [34]. The high genetic heterogeneity of glioma, and the poor response to targeted treatment with a high rate of recurrence [35, 36], makes it hard to treat patients with glioma. To better understand the mechanisms underlying glioma, researchers are actively investigating the molecular and genetic processes linked with the different types of gliomas and patient survival or risk of recurrence.

**Figure 2:**
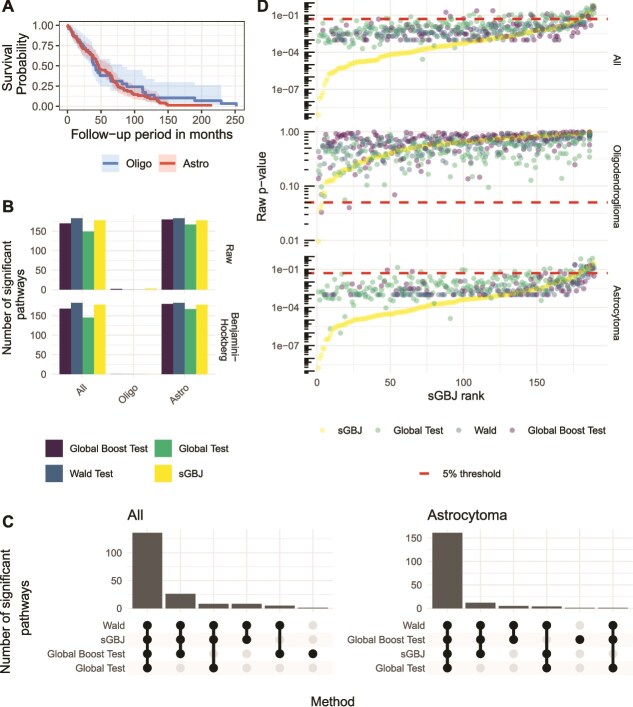
Panel A shows Kaplan–Meier curves for Rembrandt patients, stratified by glioma type. Panel B displays the number of significant pathways identified by the four methods (sGBJ, global boost test, Wald test, and global test) for astrocytoma, oligodendroglioma, and the combined cohort with all patients, at the 5% threshold using either raw $P$-values or adjusted $P$-values for multiple testing with the Benjmaini–Hochberg procedure. Panel C illustrates the agreement between the significant pathways identified by the different methods. Panel D plots the raw $P$-values against the ordered ranks of sGBJ for all four methods, with a 5% significance threshold. $P$-values equal to zero were excluded for clarity on the log scale.

Rembrandt is one of the largest public datasets on glioma, featuring clinical and genomic data on 671 patients with any types of gliomas (based on the 2007 WHO classification) collected across 14 institutions. This bioinformatic platform for brain cancer research is accessible from the Georgetown University’s G-DOC System [27, 37, 38]. For our application, we restricted our comparison to patients with either one of the two main glioma types: (i) astrocytoma and (ii) oligodendroglioma. We included all such patients from Rembrandt, for whom both tumor gene expression (as measured by micro-array) and overall survival follow-up was available. Of note, Glioblastoma patients were excluded to avoid too large genetic variation across compared glioma types. Table 1 presents the different characteristics of the total 154 patients included, and Panel A of Fig. 2 displays the corresponding Kaplan–Meier curves stratified on the glioma type. We observe that astrocytoma and oligodendroglioma patients present similar survival curves.

**Table 1:** Characteristics of astrocytoma and oligodendroglioma patients included from the Rembrant dataset

	Astrocytoma (108)	Oligodendroglioma (46)
**Age <40 yo** n (%)	52 (48 %)	22 (48 %)
**Male** n (%)	71 (66 %)	23 (50%)
**Death** n (%)	85 (79 %)	34 (74 %)
**Median follow-up period in months Median (IQR)**	37 (15 ; 66)	32 (16 ; 48)

We studied the association between patient survival and pathway gene expression, both overall and specifically for the two types of glioma: astrocytoma and oligodendroglioma. We investigated the C6 collection, from MSigDB [6]. It contains 189 gene sets related to cellular pathways that are often found to be disregulated in oncogenic studies. We used the following hazard equation to link the recurrence time with gene expression within a gene set of interest:


(9) \begin{eqnarray*}& \lambda \left( t| \mathbf{G}_{i}, \mathbf{Z}_{i} \right) = \lambda_{0}(t)\, \exp\left(\mathbf{G}_{i}^{\mathbf{\beta}} + \mathbf{Z}_{i}^{\mathbf{\gamma}}\right),\end{eqnarray*}

with $\lambda _{0}(t)$ the baseline hazard function, $G_{ij}$ the gene expression of the gene $j$ for the patient $i$, $\beta _{j}$ the associated effect, and $Z_{il}$ the values of the covariate $l$ (i.e. age or sex) with $\gamma _{l}$ the associated effect. The null hypothesis tested by the method is that all $\beta _{j}$ are zeros within a gene set. Of note, Wald test was not adjusted on age and sex given the lack of implementation of adjusted Wald test.

No pathways were identified as significantly associated with survival in oligodendroglioma patients (after multiple testing correction). However, for astrocytoma patients, the Wald test identified 96.8% of pathways as significant after applying the Benjamini–Hochberg correction for multiple testing [39]. Most methods yielded similar results, except for the global test, which identified only 76.7% of significant pathways compared with 88.9% for the global boost test, 94.2% for sGBJ, and 96.8% for the Wald test, as shown in Panel B of Fig. 2. The methods demonstrated good agreement in identifying significant pathways, as illustrated in Panel C of Fig. 2.

Panel D of Fig. 2 presents the $P$-values computed by the different methods. Both the sGBJ and global test methods use asymptotic $P$-values (note that the *GlobalTest*  R package does not support permutation $P$-values when using a Cox model with covariates). As a result, these methods have greater accuracy compared with those relying on 1000 permutations, where the lowest nonzero $P$-value achievable is 1/1001. While this limitation in permutation number does not significantly affect oligodendroglioma patients (as their $P$-values are well above $10^{-3}$), it has a minor impact on astrocytoma patients. This limitation underscores the importance of precise $P$-value estimation, particularly in multiple testing contexts [40]. $P$-values from the Cauchy and harmonic mean methods were not computed due to their failure to control the Type I error rate.

### Progression-free survival in breast cancer

Breast cancer is the most common cancer, with the number of global deaths predicted to reach 11 million per year by 2030 [41]. Survival is highly impacted by the presence of metastasis [42], which justifies the search for genes or pathways associated with either death or metastasis.

We analyzed gene expression and clinical data from the Netherlands Cancer Institute. Primary breast carcinomas data were available in the R package *breastCancerNKI* on bioconductor [43], originally reported by Van De Vijver *et al*. [28] and later reanalyzed by several teams [18, 19]. This data set comprises 24 481 gene expressions measured by Agilent technology and 337 samples. We removed 42 patients with missing phenotype data. Following Gao *et al*. [44], we removed genes with >10% missing data and imputed the remaining missing data using nearest neighbor averaging [45]. Gene expression was further standardized across samples through quantile normalization (as implemented in the *limma* bioconductor package) [46]. The median follow-up period for the 295 remaining patients was 7.2 years, and two events of interest were recorded: the appearance of metastasis and death. We studied metastasis-free survival, i.e. a composite outcome considering time to either metastasis or death, and its potential association with specific gene sets or pathways.

**Figure 3:**
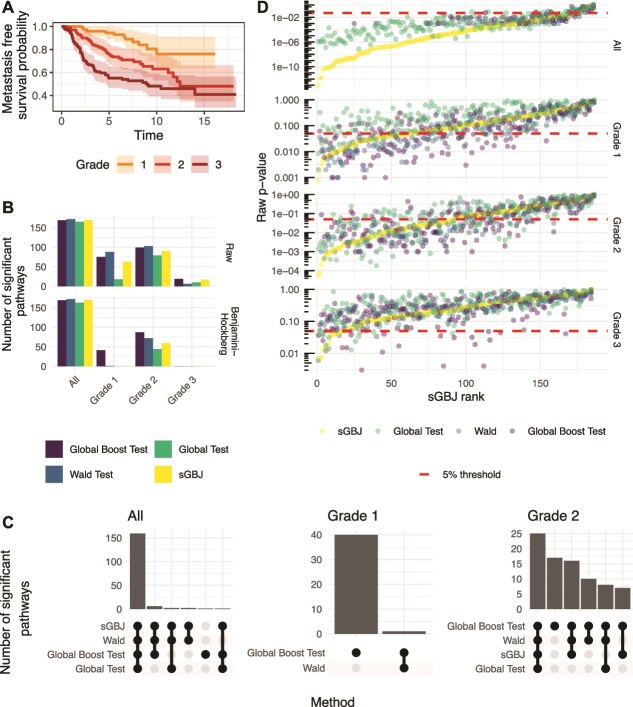
Panel A shows Kaplan–Meier curves for breast cancer patients, stratified by cancer grade. Panel B displays the number of significant pathways identified by the four methods (sGBJ, Global Boost Test, Wald Test, and Global Test) for each grade, and the combined cohort with all patients, at the 5% threshold using either raw $P$-values or adjusted $P$-values for multiple testing with the Benjmaini–Hochberg procedure. Panel C illustrates the agreement between the significant pathways identified by the different methods. Panel D plots the raw $P$-values against the ordered ranks of sGBJ for all four methods, with a 5% significance threshold. $P$-values equal to zero were excluded for clarity on the log scale.

Table 2 presents the different characteristics of the patients included, and Panel A of Fig. 3 displays the corresponding Kaplan–Meier curves.

**Table 2:** Characteristics of the 295 patients included in our metastasis free survival analysis of data from Van De Vijver *et al*. [28]

	Overall (260)
**Age in years** Median (IQR)	44 (40 ; 49)
**Grade**	
1	75 (25 %)
2	101 (34 %)
3	119 (40 %)
**Event—metastasis or death** n (%)	106 (36 %)
**Median follow-up period in years Median (IQR)**	7.2 (5.3 ; 10.3)

We studied the association between the metastasis-free survival and gene set expression adjusted for age [47], with and without stratifying on severity grade using the following Cox model:


(10) \begin{eqnarray*}& \lambda(t,\mathbf{G}_{i})=\lambda_{0}(t) \exp(\mathbf{G}_{i}^{\mathbf{\beta}} + \gamma \, \mathrm{Age}_{i}),\end{eqnarray*}

where $\lambda _{0}(t)$ is the baseline hazard function, $G_{ij}$ represents the gene expression of gene $j$ for patient $i$, and $\beta _{j}$ is the associated effect. The null hypothesis tested by the method assumes that all $\beta$ values are null. Of note, Wald test was not adjusted on age and sex given the lack of implementation of adjusted Wald test.

We investigated 186 pathways from the KEGG database [48] corresponding to the version v2.5 of the “Canonical Pathway” (CP) collection from the C2 (‘curated gene sets’) category of mSigDB (Molecular Signature Database [49]. Among these pathways, 92.5%, 91.4%, 90.9%, and 87.6% were identified as significant after applying the Benjamini–Hochberg correction for the Wald test, sGBJ, global boost test, and global test, respectively. Only the global boost test and Wald identified significant pathways among the 75 Grade 1 patients. No method identified significant pathways among the 119 Grade 3 patients, as shown in Panel B of Fig. 3. Panel C of Fig. 3 illustrates a general agreement between the methods, with the exception of the global test. As discussed in Section Survival analysis in glioma subtypes, asymptotic methods such as sGBJ and the global test can estimate $P$-values below 1e-3, a capability not achievable with the other methods using only 1000 permutations. $P$-values from the Cauchy and harmonic mean methods were not computed due to their failure to control the Type I error rate.

## Discussion

Gene set analysis methods for time-to-event data are relatively limited, and there is minimal guidance on selecting the most appropriate approach for specific contexts. To address this gap, we developed the sGBJ method, an extension of the GBJ test, tailored for gene set analysis in survival studies. To evaluate the effectiveness of sGBJ alongside other existing methods, we conducted a comprehensive benchmarking study aimed at identifying the most suitable approach under various conditions.

Our simulation studies revealed that the sGBJ method had a stringent control of type I error, which could reduce its statistical power, especially in scenarios involving a number of genes greater than or equal to the number of observations. This issue was more pronounced compared with other methods, which generally demonstrated better power under similar conditions. Additionally, similar to Lee *et al*. [23], we identified a limitation of the global test method, which assumes that all gene effect sizes follow the same normal distribution—an assumption that may not always hold and could potentially limit the method’s effectiveness, as illustrated by our Type C scenario. The Wald test consistently produced reliable results across different settings. The global boost test excelled in scenarios where genes had large effect sizes (i.e. Type C) but showed a decrease in power compared with other methods when gene correlation was very high. In contrast to the other approaches, both the Cauchy and harmonic mean approaches exhibited unreliable Type-I error control in the simulations. Previous studies have reported similar behavior, showing that these methods may fail to properly control Type I error under moderate dependence between tests [50–53].

Even though sGBJ demonstrates lower performance when the number of genes is high compared with the number of individuals, its power is comparable with other methods in most scenarios. Furthermore, in two real-world examples, this method identified a number of significant pathways on par with the other methods—while always maintaining type-I error control in simulation studies. Therefore, while sGBJ does not outperform previous methods, it appears as a sound and competitive alternative when the number of genes is smaller than the number of observations. Currently, the estimation of the covariance matrix of gene effects relies on permutation. Alternative approaches, such as shrinkage estimators, could potentially improve the performance of this method [54]. However, their application is not straightforward, as one needs to estimate the covariance matrix of the Wald statistics (and not that of the gene expressions themselves). Future work may explore this direction in an effort to further improve the sGBJ method.

Our findings complement those reported by Lee *et al*. [23], who compared the global test, Wald test, and global boost test. Their study found high power across all three methods; however, their simulation settings aligned with the global test’s assumption that all gene effects (i.e. $\beta$) are sampled from the same distribution. This likely contributed to the better performance of the global test in their simulations, particularly in contrast to our Type C scenario. In their real-world analysis of ovarian cancer data, Lee *et al*. [23] observed that the global test, the Wald test, and the global boost test identified 12, 6, and 8 significant pathways, respectively. In our real-world data analysis, Wald test, global boost test, and sGBJ consistently demonstrated high statistical power on both the Rembrandt and breast cancer datasets, while global test identified less significant genes. However, the current implementation of the Wald test does not allow adjustment for confounding factors, which may prevent its use in routine practice. Future research could further explore the replicability of these findings in additional real-world applications and under more challenging conditions, for instance when extreme outliers occur in gene effects or when batch effects are present.

In Rembrandt, no oncogenic pathways were associated with survival in oligodendroglioma, whereas nearly all were significant in astrocytoma. This surprising result may be explained by several factors: (i) astrocytomas and oligodendrogliomas are driven by distinct molecular mechanisms. For instance, astrocytomas more frequently harbor TP53 and ATRX alterations, whereas oligodendrogliomas are defined by 1p/19q codeletion and exhibit greater genomic stability [55–57]. (ii) The oncogenic pathways from MSigDB show substantial gene overlap. Remarkably, just seven survival-associated genes collectively appear in nearly half of the pathways, indicating that pathway-level signals can be strongly influenced by a small number of key genes. (iii) The oligodendroglioma subgroup contains fewer patients, reducing statistical power and contributing to the absence of significant pathways after multiple-testing correction. (iv) Pathway expression may influence glioma subtype; by analyzing subgroups separately, we condition on a mediator (astrocytoma/oligodendroglioma status), thereby blocking indirect causal effects of pathway expression on survival and focusing only on the direct effect. This conditioning may contribute to the unexpected subgroup-specific results. A formal mediation analysis would be required to disentangle these effects, which is beyond the scope of this study. Similarly, in breast cancer, many KEGG pathways were found significant in the Grade 2 group, while few were significant in Grade 1 or Grade 3 groups. This pattern may reflect the same factors: high pathway gene overlap, reduced power in smaller subgroups, and conditioning on tumor grade as a mediator, which affects the observed pathway–survival associations.

In practice, the choice of gene set test depends on data characteristics. When strong power and Type I error control are required in typical correlation settings, the Global boost test or Wald test are preferred, although in the current implementation the Wald test does not allow adjustment for confounding factors. In scenarios with very high gene correlation, the Global boost test may lose power, making sGBJ a more robust option if the number of observations is large relative to the number of genes. The Global test generally performs well in simulations but may have lower power in real-world applications. Methods based on $P$-value combinations, such as Cauchy and Harmonic Mean, are not recommended due to poor Type I error control.

The adaptation of sGBJ method to the time-to-event context is implemented in an R package available on the CRAN at https://CRAN.R-project.org/package=sGBJ. Code for this analysis is available at https://github.com/thomasferte/sGBJ_computation and on Zenodo [58].

**Key Points**

- sGBJ is a gene set analysis method for survival data that accounts for gene–gene correlation.
- sGBJ extends the Generalized Berk–Jones statistic within a Cox proportional hazards model framework.
- sGBJ strictly controls type I error, which may reduce its statistical power, particularly in high-dimensional settings.
- The Global Boost test generally exhibit higher power across various scenarios.
- In the presence of high correlation between genes, sGBJ represents the most robust option as long as the number of genes is low relatively to the number of observed samples.

## Supplementary Material

supplementary_sGBJ_bbag262

## Data Availability

The adaptation of sGBJ method to the time-to-event context is implemented in an R package available on the CRAN at https://CRAN.R-project.org/package=sGBJ. Analysis code is available at https://github.com/thomasferte/sGBJ_computation and on Zenodo [58]. Data for Rembrandt study are available at https://www.ncbi.nlm.nih.gov/geo/query/acc.cgi?acc=GSE108474. Breast Cancer data are available at [43].
