# Supplementary material for: Gene set analysis for time-to-event outcome: comparison of a new approach based on the generalized Berk–Jones statistic with existing methods in presence of intra gene-set correlation
Source: Brief Bioinform. 2026 May 31;27(3):bbag262. doi: 10.1093/bib/bbag262 (PMC13222529; doi:10.1093/bib/bbag262)
Supplement: supplementary_sGBJ_bbag262 [file supplementary_sgbj_bbag262.pdf]

Gene Set Analysis for time-to-event outcome:comparison of a new approach based on the Generalized Berk–Jones statistic with existing methods in presence of intra gene-set correlation

Supplementary

2026-03-18

Contents

1 Simulation Settings from Real Data **1**

1.1 Proportion of Significant Genes 1

1.2 Gene expression variance 2

1.3 Gene expression correlation 2

1.4 Beta from Cox model 4

2 QQ-plot main analysis **6**

3 Sensitivity Analysis **7**

3.1 High correlation setting 7

3.2 Varying proportion of significant genes 7

3.3 Large number of observations 8

4 Estimation of covariance matrix by sGBJ **10**

1 Simulation Settings from Real Data

This section aims to identify realistic simulation settings using the Rembrandt and Breast Cancer datasets.

1.1 Proportion of Significant Genes

As shown in Figure 1, the proportion of significant genes is approximately 45% for the Rembrandt dataset and 20% for the Breast Cancer dataset.

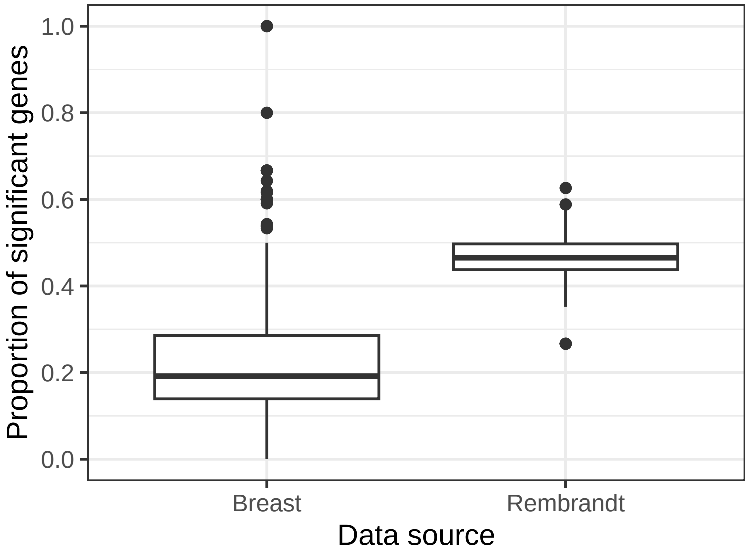

Figure 1: Proportion of significant genes in each pathway for each data source.

1.2 Gene expression variance

Gene expression variance was studied in each data source. The median was approximately 0.3 and 0.09 in Rembrandt and Breast cancer data respectively. For simulations, we use an intermediate variance of 0.2 (Figure 2).

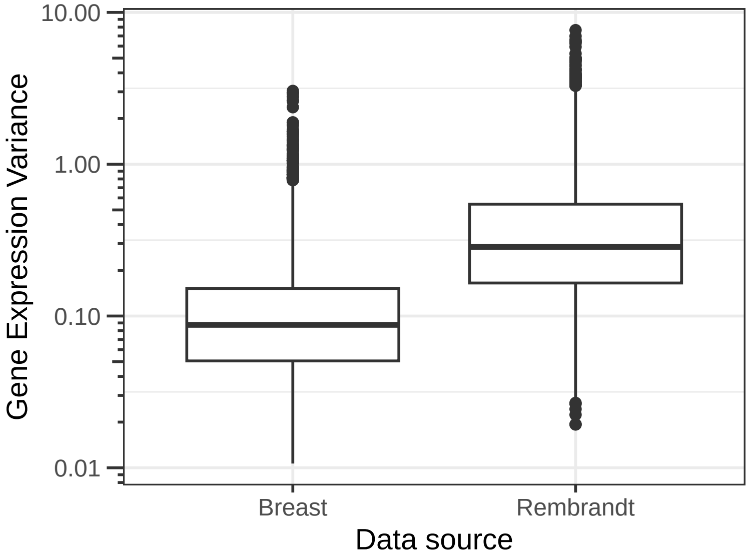

Figure 2: Gene expression variance for each data source.

1.3 Gene expression correlation

Gene expression correlation was evaluated on each pathway of each data source. Correlation was evaluated overall and stratified by gene significance. Figure 3 indicates that an overall gene correlation following a non-standard beta distribution(20, 20, min = -1, max = 1) is realistic for both Rembrandt and Breast cancer

data. This is denoted as case I. When stratified by gene significance, figure 4 outlines that a non-standard beta distribution(10, 10, min = -1, max = 1) for significant genes and non-standard beta distribution(25, 25, min = -1, max = 1) for non-significant genes is realistic. This is denoted as case II.

A simpler simulation setting considering 0.2 correlation between significant genes and 0 correlation between non-significant genes was also considered.

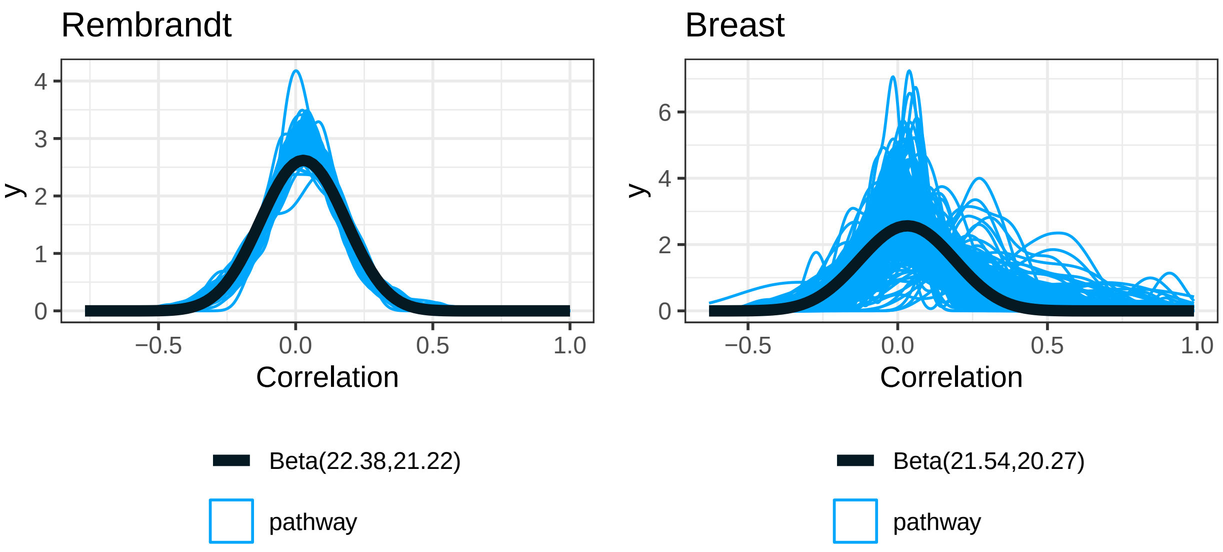

Figure 3: Gene expression correlation for each pathway in each data source.

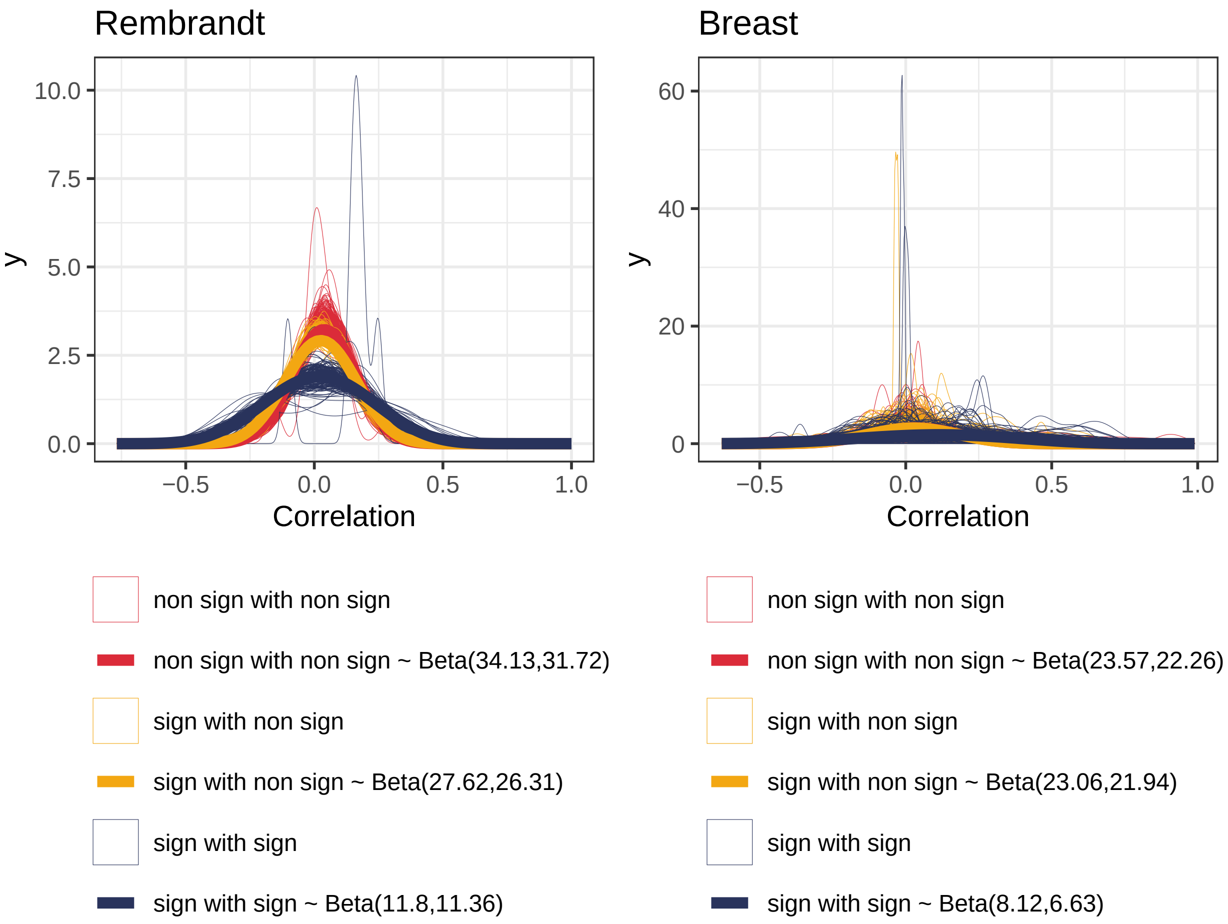

Figure 4: Gene expression correlation for each pathway in each data source stratified by gene significance.

1.4 Beta from Cox model

Beta coefficient distributions, both overall and stratified by gene significance, were examined in the two datasets. Figure 5 indicates that the overall distribution of effect sizes is well approximated by a normal distribution with mean 0 and standard deviation 0.4; we refer to this setting as Type A. Figure 6 further shows that, in the Rembrandt dataset, half of the gene effects follow a normal distribution with mean -0.4 and standard deviation 0.2, while the other half follow a normal distribution with mean 0.4 and standard deviation 0.2; we denote this as Type B. In contrast, the Breast Cancer dataset is characterized by a wider separation, with half of the effects following a normal distribution with mean -0.8 and standard deviation 0.4, and the other half following a normal distribution with mean 0.8 and standard deviation 0.4; this is referred to as Type C.

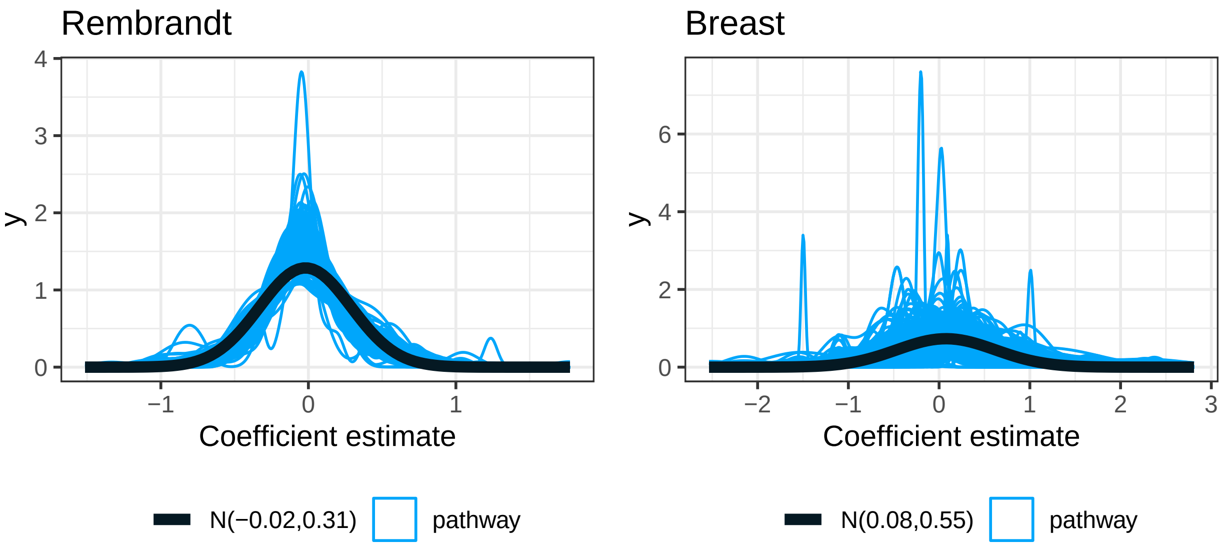

Figure 5: Beta coefficient distribution for each data source.

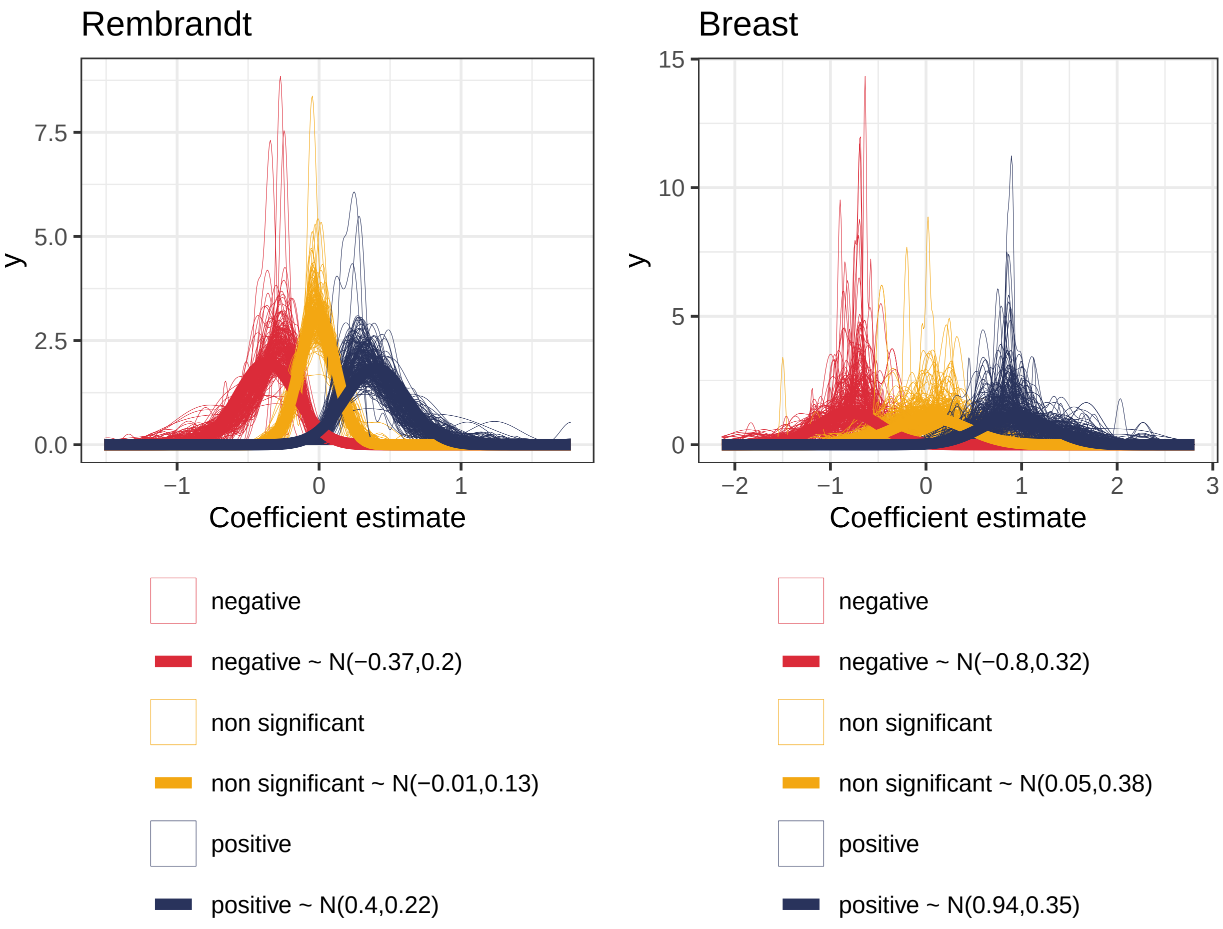

Figure 6: Beta coefficient distribution for each data source stratified by gene significance.

2 QQ-plot main analysis

Figure 7 presents quantile–quantile (QQ) plots of the p-values obtained with the different methods, compared to the uniform distribution. The results indicate that the Harmonic Mean and Cauchy methods tend to under-control the Type I error, whereas sGBJ is overly conservative. Notably, sGBJ exhibits an unexpectedly large number of p-values close to 1 compared with the theoretical distribution; however, this behavior is consistent with its conservative nature and reflects an excess of non-significant p-values.

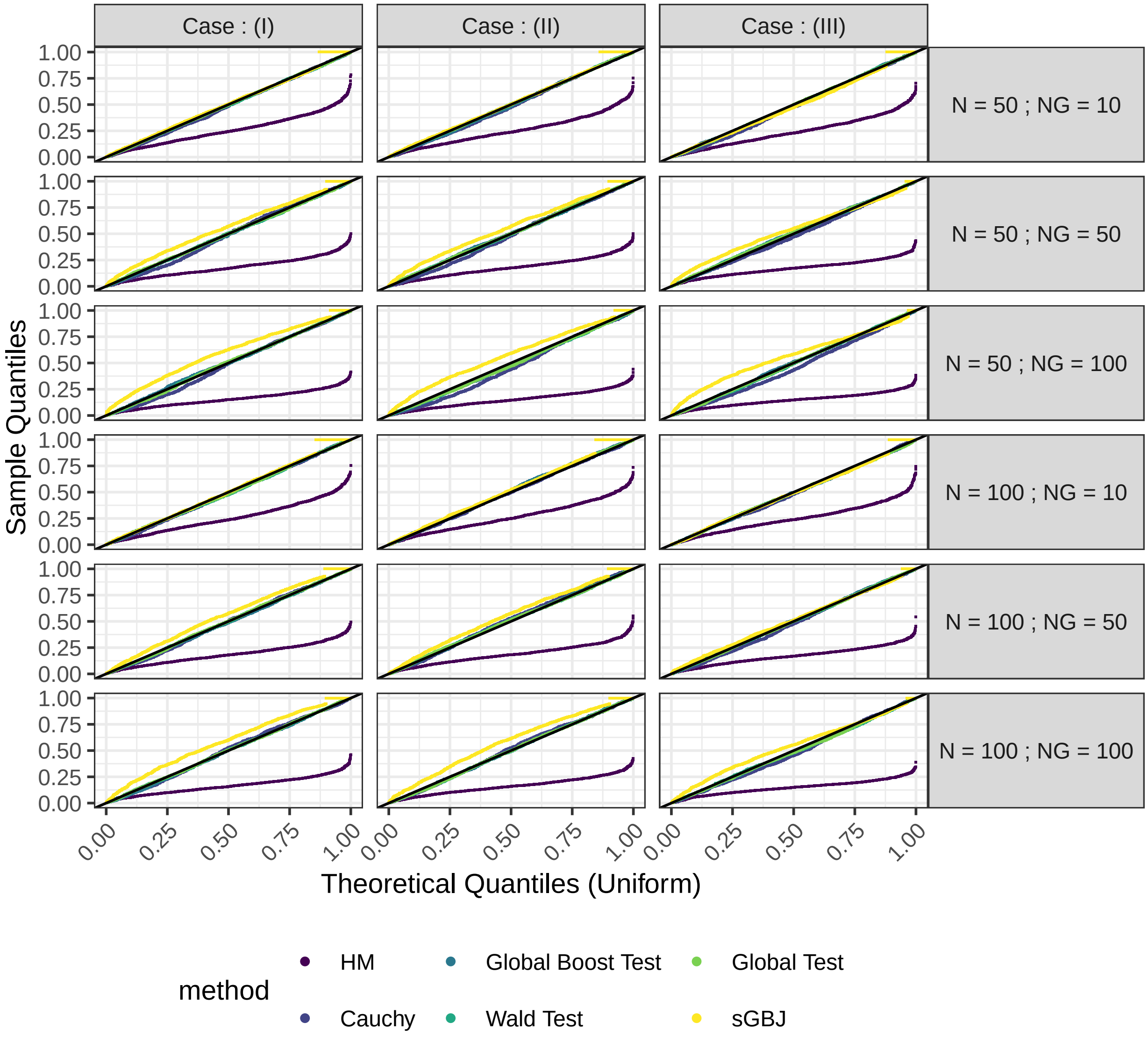

Figure 7: Quantile–quantile (QQ) plots of p-values for the different methods under the null hypothesis, compared with the uniform distribution. Well-calibrated methods are expected to align with the 45-degree diagonal.

3 Sensitivity Analysis

3.1 High correlation setting

Figure 8 presents Case IV, in which significant genes are correlated with each other with a correlation of 0.9. Panel A shows that power is very similar across the different methods, except for the Global Boost Test, which exhibits a decrease in power. Panel B shows a similar failure of Type I error control for the Harmonic Mean and Cauchy methods as observed elsewhere in the paper.

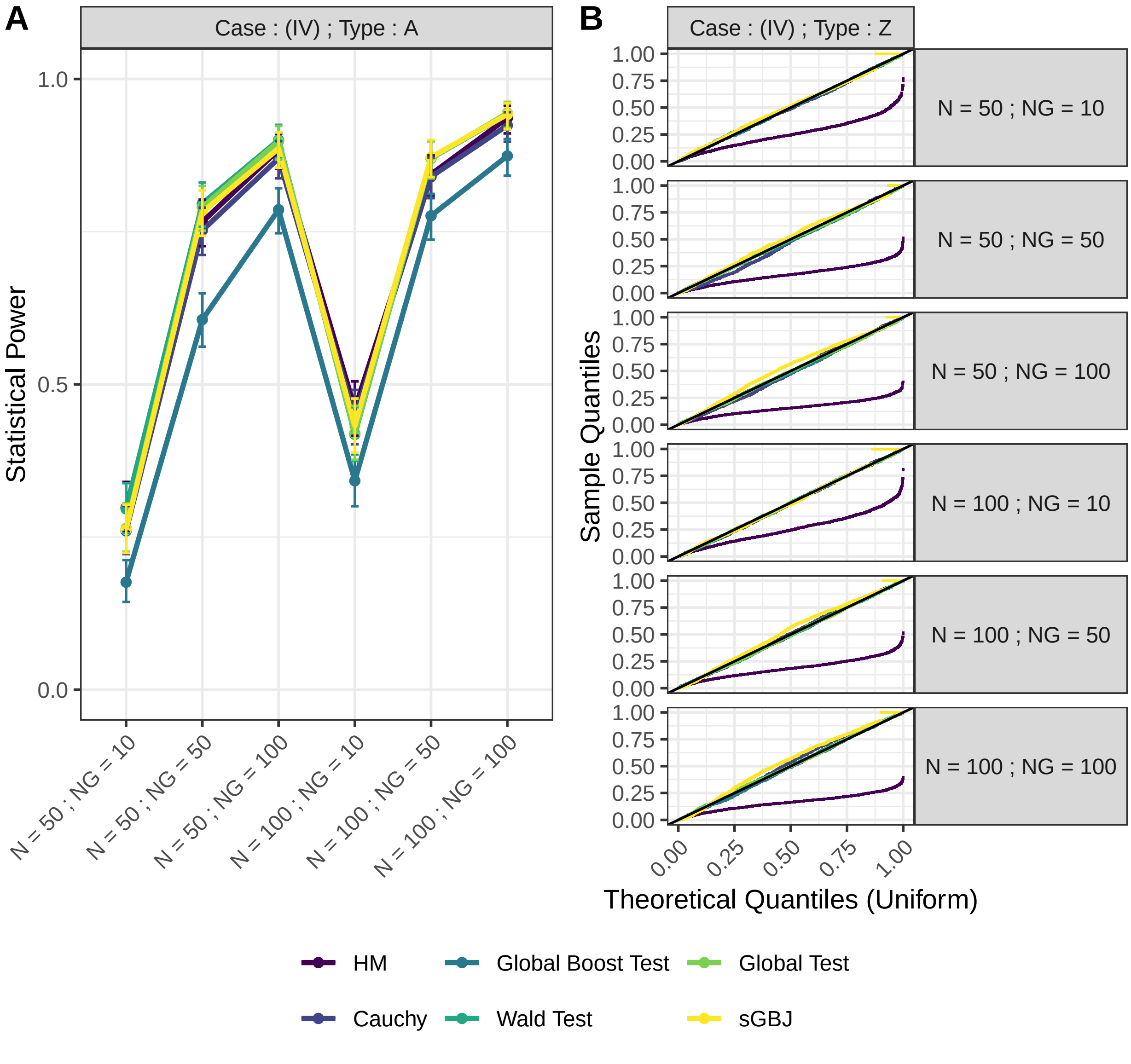

Figure 8: Panel A shows the statistical power of the different methods in Case IV, Type A. Panel B shows p-values for each method compared with the uniform distribution under the Type-Z (no association) setting; well-calibrated methods align with the 45-degree diagonal.

3.2 Varying proportion of significant genes

Figure 9 shows the behavior of the different methods as the proportion of significant genes varies. The results indicate that the conservative Type I error correction of sGBJ has a stronger impact as the proportion of

significant genes decreases. The other methods exhibit similar performance. The Cauchy and Harmonic Mean methods still fail to control the Type I error.

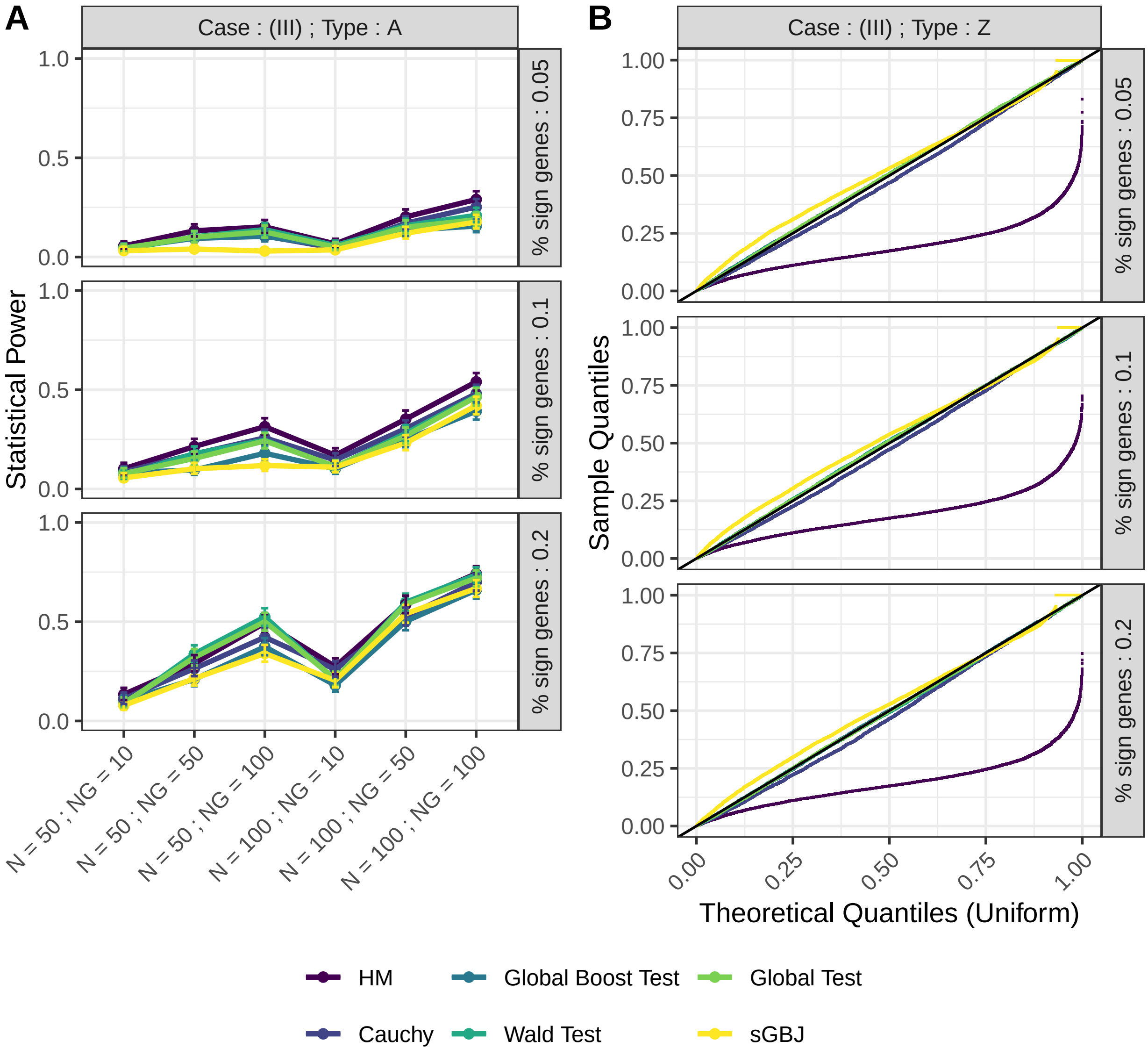

Figure 9: Panel A shows the statistical power of the different methods in Case III, Type A with various proportion of significant genes. Panel B shows p-values for each method compared with the uniform distribution under the Case III, Type-Z (no association) setting; well-calibrated methods align with the 45-degree diagonal.

3.3 Large number of observations

Figure 10 illustrates the behavior of the different methods as the number of observations increases up to 1,000. The results indicate that the conservative Type I error correction of sGBJ becomes less problematic when the number of observations is large relative to the number of genes. This suggests that the limitation of sGBJ is primarily a dimensionality issue: the method does not perform well under a high p/n ratio, rather than being affected by the absolute number of genes. In contrast, the Cauchy and Harmonic Mean methods continue to fail to adequately control the Type I error.

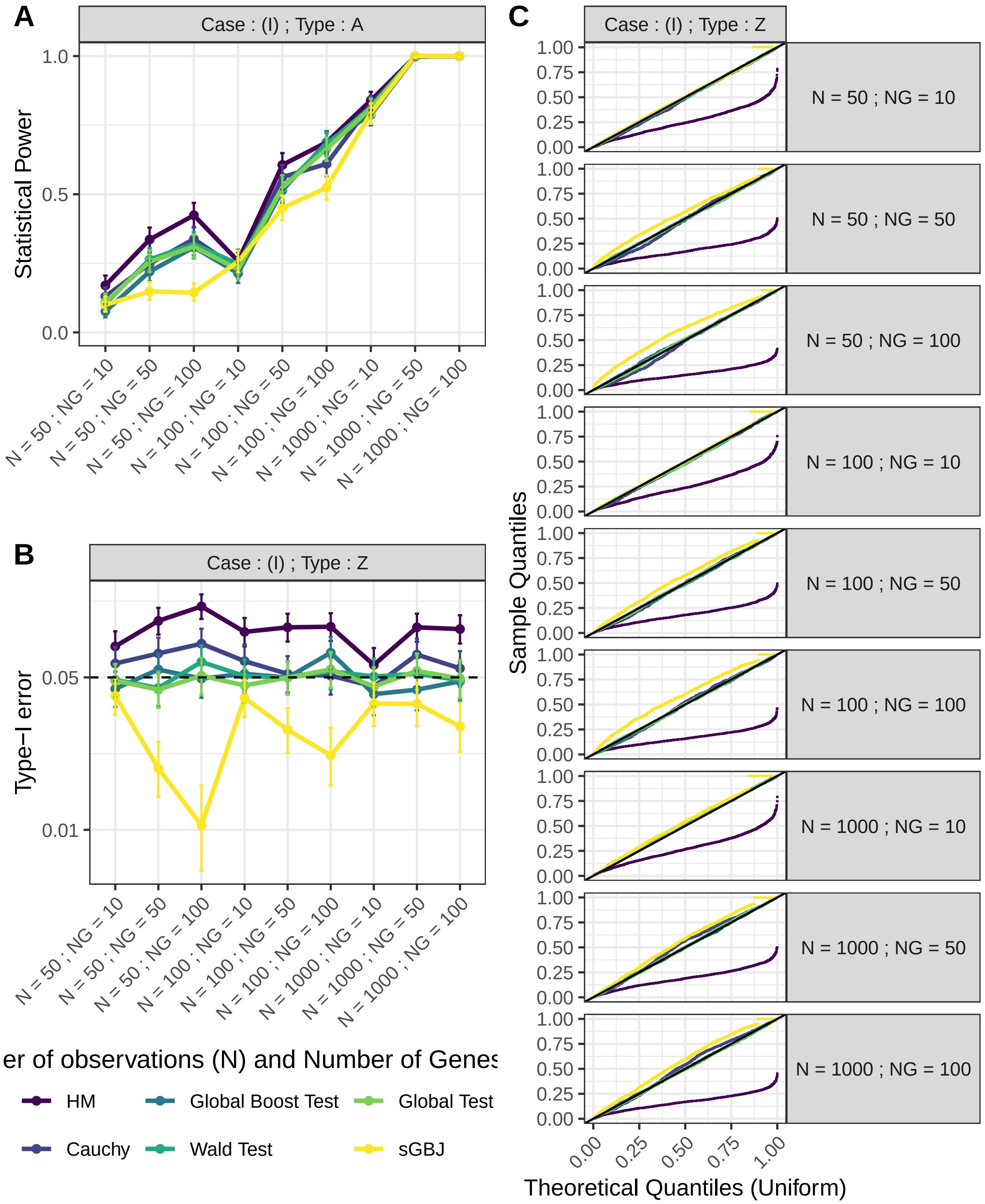

Figure 10: Panel A displays the statistical power of the different methods in Case I, Type A, as the number of observations increases from 50 to 1,000. Panel B shows the corresponding Type I error rates for the different methods, with a nominal target level of 0.05. Panel C presents quantile–quantile plots of p-values for each method under the Case III, Type Z (no association) setting; well-calibrated methods follow the 45-degree diagonal.

4 Estimation of covariance matrix by sGBJ

Figure 11 shows the Frobenius distance between the true gene-effect covariance matrix and the matrix estimated by sGBJ for Case (I), Type B. The results indicate a substantial improvement in covariance estimation when the number of permutations increases from 8 to 200. Increasing the number of permutations further to 1,000 and 5,000 yields little additional improvement. These conclusions hold regardless of the number of genes or observations.

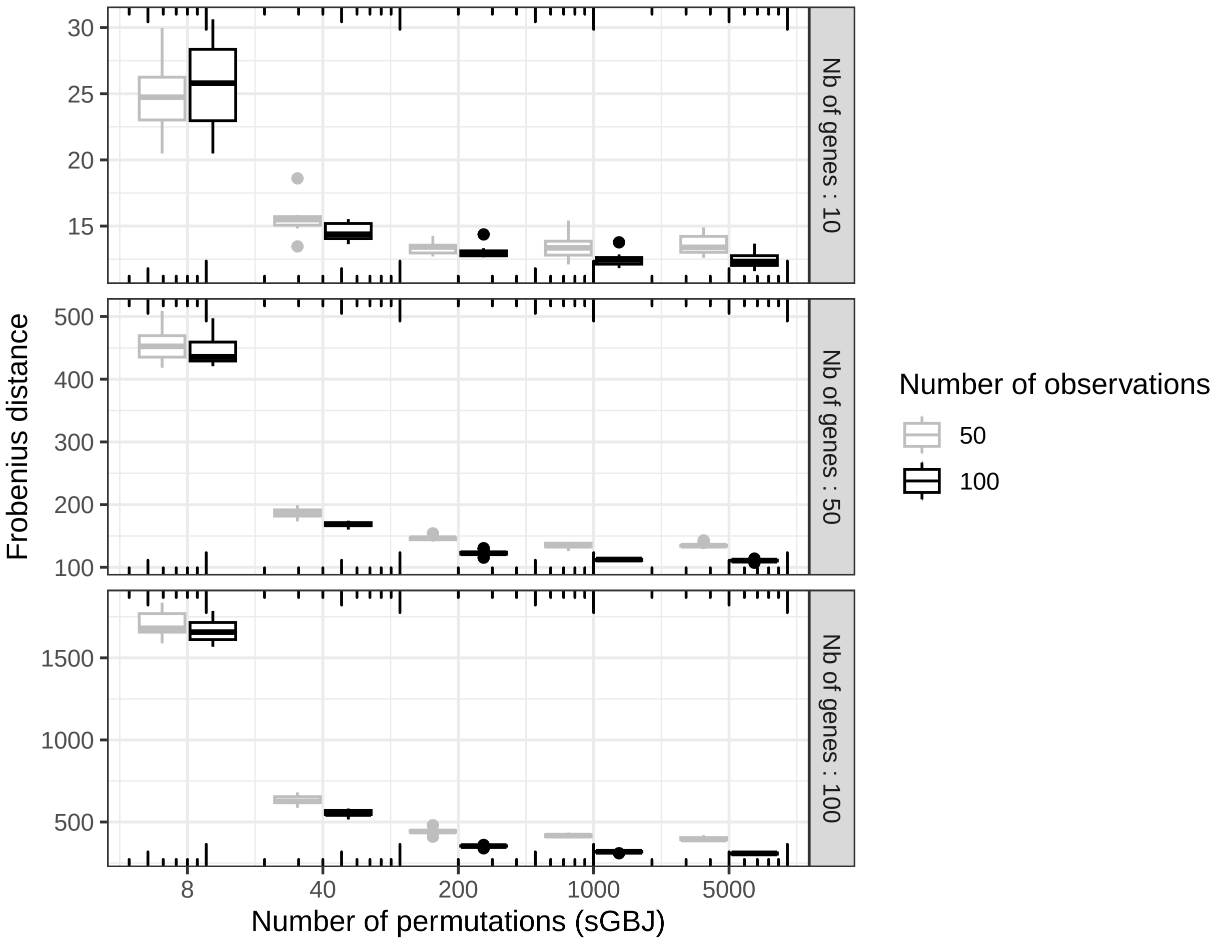

Figure 11: Frobenius distance between the true and sGBJ-estimated gene effect covariance matrices as a function of the number of observations, number of genes, and number of sGBJ permutations. Experiment on Case (I), Type : B.
